# Examining the Factor Structure of the Home Mathematics Environment to Delineate Its Role in Predicting Preschool Numeracy, Mathematical Language, and Spatial Skills

**DOI:** 10.3389/fpsyg.2020.01925

**Published:** 2020-08-06

**Authors:** David J. Purpura, Yemimah A. King, Emily Rolan, Caroline Byrd Hornburg, Sara A. Schmitt, Sara A. Hart, Colleen M. Ganley

**Affiliations:** ^1^Department of Human Development and Family Studies, Purdue University, West Lafayette, IN, United States; ^2^Department of Human Development and Family Science, Virginia Tech, Blacksburg, VA, United States; ^3^Department of Psychology, Florida State University, Tallahassee, FL, United States

**Keywords:** home mathematics environment, mathematics, parent–child interactions, numeracy skills, mathematical language, spatial skills, preschool

## Abstract

A growing body of evidence suggests that the ways in which parents and preschool children interact in terms of home-based mathematics activities (i.e., the home mathematics environment; HME) is related to children’s mathematics development (e.g., primarily numeracy skills and spatial skills); however, this body of evidence is mixed with some research supporting the relation and others finding null effects. Importantly, few studies have explicitly examined the factor structure of the HME and contrasted multiple hypothesized models. To develop more precise models of how the HME supports children’s mathematics development, the structure of the HME needs to be examined and linked to mathematics performance. The purpose of this study was to extend prior work by replicating the factor structure of the HME (as one general HME factor and three specific factors of direct numeracy, indirect numeracy, and spatial) and using those factors to predict direct assessments of children’s numeracy, mathematical language, and spatial skills. It was hypothesized that the general HME factor would be related to each direct assessment, the direct numeracy factor would be related to both numeracy and mathematical language, and the spatial factor would be related to spatial skills. Using a sample of 129 preschool children (*M* age = 4.71 years, *SD* = 0.55; 46.5% female), a series of confirmatory factor analyses were conducted. Results diverged somewhat from prior work as the best fitting model was a bifactor model with a general HME factor and two specific factors (one that combined direct and indirect numeracy activities and another of spatial activities) rather than three specific factors as had previously been found. Further, structural equation modeling analyses suggested that, in contrast to expectations, only the direct + indirect numeracy factor was a significant predictor of direct child assessments when accounting for age, sex, and parental education. These findings provide evidence that a bifactor model is important in understanding the structure of the HME, but only one specific factor is related to children’s outcomes. Delineating the structure of the HME, and how specific facets of the HME relate to children’s mathematics skills, provides a strong foundation for understanding and enhancing the mechanisms that support mathematics development.

## Introduction

The home learning environment that parents provide for their children is an important context for the development of academic skills, including mathematics (Manolitsis et al., 2013). Children typically acquire early mathematics skills in everyday informal settings and experiences, such as interacting with parents in the home (Ginsburg, 1977; Baroody and Wilkins, 1999; Dickinson and Tabors, 2001; Melhuish et al., 2008). Emerging evidence suggests that the “home mathematics environment” (HME), a term used to describe mathematics-related activities children engage in with their parents, is a significant predictor of children’s broad mathematics skills (e.g., encompassing specific skills such as numeracy, geometric reasoning and spatial skills, patterning skills, and measurement; Blevins-Knabe and Musun-Miller, 1996; LeFevre et al., 2009; Kleemans et al., 2012; Levine et al., 2012; Niklas and Schneider, 2014; Hart et al., 2016; Zippert and Rittle-Johnson, 2018). Despite this growing body of work, few studies have explicitly examined and contrasted if there are distinct aspects of the HME for a preschool-age population or the extent to which these different aspects may uniquely predict direct assessments of children’s mathematics outcomes. Further, studies on the HME typically only include child skills such as numeracy and spatial skills because they are two of the strongest predictors of broader mathematics skills development (Nguyen et al., 2016; Mix, 2019). Other domains such as mathematical language, an important foundation for numeracy development in young children (Purpura and Logan, 2015), has not previously been linked to the HME. The present study addressed these limitations by (1) comparing multiple factor structures of the HME in a sample of preschoolers, and (2) examining the extent to which different HME factors predict children’s numeracy skills, mathematical language skills, and spatial skills, which are key abilities that predict more advanced mathematics development (Aunola et al., 2004; Toll and Van Luit, 2014; Verdine et al., 2014; Nguyen et al., 2016).

### Structure of the Home Mathematics Environment

The HME has been identified as a critical context where young children develop their early mathematics skills (Blevins-Knabe and Musun-Miller, 1996; Gunderson and Levine, 2011; Anders et al., 2012; Blevins-Knabe, 2012; Manolitsis et al., 2013; Niklas et al., 2016). For example, children whose parents used more number talk (e.g., counting and labeling large sets of objects at home) with them when they were between 14 and 30 months old had higher mathematics skills than their peers at age three (Gunderson and Levine, 2011). In another study, when families attended meetings that provided them with information on the importance of the HME and the principles of counting, the frequency of parent–child engagement in mathematics-related activities increased and children had higher mathematics skills than children from families who did not attend those sessions (Niklas et al., 2016). Yet, understanding of the HME is still in development (Elliott and Bachman, 2017), particularly in terms of what are its unique aspects and how these aspects individually relate to young children’s mathematics skills.

The HME has been conceptualized in a variety of ways, but most work has typically focused on the numeracy aspects of the HME (LeFevre et al., 2009; Thompson et al., 2017). Specifically, these studies focus on two components of the construct: direct and indirect numeracy activities (LeFevre et al., 2009, 2010; Manolitsis et al., 2013; Skwarchuk et al., 2014; Thompson et al., 2017). Direct activities are specific experiences that parents provide for their children that explicitly teach quantitative skills (e.g., counting, reading number storybooks). Indirect activities consist of a broader range of everyday experiences that implicitly teach quantitative skills (e.g., measuring ingredients, talking about money). Direct numeracy and indirect numeracy activities have also been called formal numeracy and informal numeracy activities, respectively (Manolitsis et al., 2013).

More recent work has pointed to the direct and indirect numeracy environments as being just two components of a broader HME, which also includes non-numeracy components such as the spatial environment (Dearing et al., 2012; Hart et al., 2016) and patterning environment (Zippert and Rittle-Johnson, 2018). For example, Dearing et al. (2012) proposed an alternative model to the direct numeracy and indirect numeracy structure of the HME that consisted of two factors: numeracy activities and spatial activities. Spatial activities included experiences that involve the perception of objects in space (e.g., drawing maps, measuring objects, building, playing with puzzles). In a more recent study that also considered spatial activities, Hart et al. (2016) tested a range of plausible models of the HME, that included both the ‘direct vs. indirect’ and ‘numeracy vs. spatial’ models, and also included testing bifactor models. Bifactor models allow the item variance to be partitioned into that which goes with a full general factor, and that which should be separated into specific factors. In this context, they found that that the best fit was a bifactor model that consisted of a general factor (general HME; this factor accounted for the common variance from across all the aspects of the HME) and three specific factors (direct numeracy environment, indirect numeracy environment, and spatial environment; these factors included the variance specific to each component after removing the common variance shared across all items).

Beyond a few studies, empirical evidence explicitly evaluating the factor structure of the HME is relatively limited. Some studies have assumed the HME to be a unidimensional construct (Blevins-Knabe and Musun-Miller, 1996) or identified a unidimensional construct through exploratory factor analysis (Blevins-Knabe and Musun-Miller, 1996; Kleemans et al., 2012). Other studies have shown the HME is multi-dimensional through exploratory factor analyses (LeFevre et al., 2009, 2010; Dearing et al., 2012; Manolitsis et al., 2013) or by using confirmatory factor analysis to test the model fit of one potential model (Van Hoof et al., 2020). However, only Hart et al. (2016) have explicitly contrasted multiple potential factor structures to examine the best-fitting factor structure. Examining the structure of the HME is important for understanding the extent to which a broad indicator of the HME best represents the construct or whether the construct is comprised of distinct, but related components. Further, understanding the structure of the HME is critical for identifying if specific components of the HME are differentially related to mathematics outcomes—and thereby, if more complex models of how the home environment potentially impacts development of specific mathematics skills are needed. There is a critical need to further evaluate the structure and test these potential models in a new sample to provide further empirical evidence for the structure of the HME. In doing so, we can better clarify if there are different aspects of the HME and what characterizes these aspects.

### Relations Between the Home Mathematics Environment and Preschool Mathematics Skills

One of the core purposes of understanding the structure of the HME is to then link it to children’s performance on measures of mathematics skills—in order to develop more precise models of which aspects of the home environment may support children’s mathematics development. Importantly, children’s early mathematics skills are not a unitary construct; they encompass a broad range of concepts including numeracy, geometric reasoning and spatial skills, patterning, and measurement (Milburn et al., 2019)—though, most empirical work on early mathematics has focused on aspects of numeracy (Methe et al., 2011) and spatial skills (Mix and Cheng, 2012) because they are most predictive of long-term mathematics development (Nguyen et al., 2016; Mix, 2019). Paralleling this work, studies of the HME also primarily focus on children’s numeracy skills (e.g., counting, numerical relations, and operations; Purpura and Lonigan, 2013). However, some work has also explicitly examined the unique effects that spatial activities within the HME may have on children’s mathematics outcomes (Dearing et al., 2012; Zippert and Rittle-Johnson, 2018). Furthermore, no work has evaluated the relation between the HME and mathematical language—a construct that appears to underlie both numeracy (Purpura et al., 2011) and spatial skills (Casasola et al., 2020). It is important to note that, regardless of which aspect of children’s performance is considered, the literature linking the HME to mathematics skills is somewhat mixed.

#### Numeracy

Most of the work linking the HME to numeracy skills has focused on the direct numeracy vs. indirect numeracy structure of HME and found that direct numeracy activities are a more consistent predictor of numeracy skills than are indirect numeracy activities (LeFevre et al., 2010; Manolitsis et al., 2013; Skwarchuk et al., 2014; Thompson et al., 2017), though there are a few exceptions (LeFevre et al., 2009; Skwarchuk et al., 2014). For example, Skwarchuk et al. (2014) found that direct numeracy and indirect numeracy activities were related to different aspects of kindergarteners’ numeracy skills, such that direct numeracy activities predicted children’s symbolic number system knowledge (e.g., knowledge of exact quantities) and indirect numeracy activities predicted children’s non-symbolic number knowledge (e.g., approximate estimation of quantities). In contrast, results of the Hart et al. (2016) bifactor model suggested the general HME factor predicted parent reports of 3-to 8-year-olds’ general mathematics skills, whereas the direct numeracy and indirect numeracy factors were not significant predictors. However, this study was limited by its use of a broad measure of parent-reported mathematics skills instead of direct assessments of children’s skills which may have introduced assessor bias into the models (parents rated both the HME and children’s performance which may have inflated the general relation among the variables). Moreover, the parent report of children’s skills included ratings of children’s “mathematics,” “numeracy,” and “spatial” skills which also may explain why a relation was found with the general HME factor rather than specific factors. If a direct assessment of children’s numeracy skills was used, it would be expected that the direct numeracy factor would be uniquely related.

#### Spatial Skills

Spatial skills have been identified as a core component and predictor of broad mathematical skills such as geometric reasoning (Verdine et al., 2014; Rittle-Johnson et al., 2019) and are malleable through intervention (Casey et al., 2008; Schmitt et al., 2018). Some evidence also suggests that parent–child engagement—specific to spatial engagement—may be associated with young children’s spatial language and spatial skills (Ferrara et al., 2011). For example, parents’ use of spatial language at home is longitudinally related to children’s use of spatial language (Pruden et al., 2011; Pruden and Levine, 2017). Further, preschool children perform better on spatial transformation tasks when they have parents who engaged with them in more puzzle play activities between the ages of two and four (Levine et al., 2012). However, there is limited work linking parent report of parent–child engagement in spatial activities with children’s spatial skills (e.g., Dearing et al., 2012; Zippert and Rittle-Johnson, 2018), particularly at the preschool level. For example, Zippert and Rittle-Johnson (2018) did not find a significant relation between parent ratings of the home spatial environment and children’s spatial skills, though neither the factor structure of the HME nor the item composition of the factor were explicitly evaluated in that study. Further, in older children (6- to 7-year olds), although Dearing et al. (2012) did examine the factor structure of the HME and separated out spatial and numerical activities, spatial activities did not predict spatial skills. Finally, in a sample that spanned the preschool and early elementary school period, Hart et al. (2016) found evidence that the home spatial environment was distinct from other aspects of the HME, but they did not find a direct relation between the factor and parent report of children’s performance. However, as similar to the limitations noted in the numeracy section, the parent report was a broad indicator of children’s mathematics skills (encompassing broad mathematics, numeracy, and spatial skills). Using a more refined measure of the home spatial environment and a direct assessment of preschool children’s spatial skills, it could be expected that a relation between the spatial environment and children’s spatial skills might be found.

#### Mathematical Language

One additional aspect of children’s early mathematics skills that has been linked to both numeracy (Purpura and Reid, 2016) and spatial skills (Casasola et al., 2020) is mathematical language (e.g., understanding words and concepts such as *many, most, few, fewest, before, after, near, far*). Mathematical language has been shown to be an important predictor of children’s mathematics development during both preschool (Purpura and Logan, 2015) and early elementary school (Toll and Van Luit, 2014). Though existing work has not directly linked parent reported HME to mathematical language, there is a growing body of evidence that would support that link. For example, recent evidence in an experimental setting suggests that parent–child interactions that are explicitly focused on teaching mathematics (direct numeracy activities) show greater amounts of math-related talk during these activities than in less direct mathematics-related activities or non-mathematics related activities (Eason and Ramani, 2020)—suggesting that when parents are engaged in direct mathematics activities, they are more likely to use (and potentially support) mathematical language compared to when they are engaged in less directed activities. Moreover, the direct activities that parents and children engage in most frequently (e.g., counting and comparing; Thompson et al., 2017) involve the numeracy skills that are most closely related to children’s mathematical language knowledge (Hornburg et al., 2018). These findings suggest that when parents engage in direct mathematics activities, there may be opportunities that not only support children’s knowledge of numeracy, but also their mathematical language skills. In terms of parent-reported HME, a recent study also revealed that parent report of direct home numeracy activities not only predicts preschoolers’ numeracy performance, but also their general vocabulary knowledge, but it does not predict specific early literacy skills (Napoli and Purpura, 2018). Given that mathematical language is an aspect of both language and mathematics, this finding would also suggest that high quality direct numeracy activities may support mathematical language; however, this relation needs to be empirically evaluated. Furthermore, as there is some evidence that parent spatial talk is linked with children’s spatial skills, there is not sufficient evidence to directly hypothesize whether or not the spatial environment, when accounting for the direct numeracy environment, will also be a predictor of mathematical language.

### Current Study

Given the questions regarding the structure of the HME and the relation of specific factors to direct assessments of children’s skills, the current study was designed to replicate and extend the Hart et al. (2016) study of the relation between the measurement structure of the HME and a parent-reported broad measure of children’s mathematics skills, by using direct assessments of specific preschool mathematics and spatial skills. We focused on a preschool-aged sample because this is an important time when young children are developing mathematics-related skills (Baroody and Wilkins, 1999), such as early numeracy skills, spatial skills, and mathematical language knowledge. Based on the results of Hart et al. (2016), it was hypothesized that the factor structure of the HME would consist of one general HME factor and three specific factors representing the direct numeracy environment, the indirect numeracy environment, and the spatial environment. We also examined the role of the HME in predicting direct assessments of preschoolers’ specific mathematics skills, including numeracy, mathematical language, and spatial skills. Expanding on the work of Hart et al. (2016) who used a combined measure of parent-reported mathematics and spatial skills, we used direct assessments of children’s numeracy, mathematical language, and spatial skills. It was expected that the bifactor model with three specific factors (direct numeracy environment, indirect numeracy environment, and spatial environment) with the broad HME factor would be replicated. It was also expected that the general HME factor would significantly predict all three direct assessments (numeracy, mathematical language, and spatial skills) because it is reflective of a broad positive HME, but given that there is greater precision of measurement with direct assessments of children’s skills than with parent report, it was also expected that the direct numeracy environment factor would be positively related to children’s numeracy skills and mathematical language and that the spatial environment factor would be positively related to children’s spatial skills.

## Materials and Methods

### Participants

Participants were recruited from 18 early childhood centers in the Midwestern region of the United States. Letters explaining the study, consent forms, and questionnaires were sent home to all parents of preschool children attending these centers. Parents of 132 preschoolers completed consent forms. Three families did not complete the home survey and, thus, were not included in this study. The 129 preschoolers (60 females and 69 males) included in the analyses were on average 4.71 years old (*SD* = 0.55), 79.1% were White/Caucasian, 2.3% were Black/African-American, 4.7% were Latino/Hispanic, 4.7% were Asian, 7.0% were other/multiracial, and 2.3% did not report race/ethnicity information. Of these families, 89.9% reported that English was the primary language spoken at home, 4.5% reported that a language other than English (e.g., Chinese) was the primary language at home, and 5.5% reported that both English and another language (e.g., Chinese, Spanish) were spoken at home. Parent education was relatively diverse with 44.2% of parents reporting less than a college degree, 28.7% reporting a 2- or 4-year degree, and 27.2% reporting a graduate degree.

### Measures

#### Home Mathematics Environment Survey

As part of a larger survey on the home environment, parents were asked to complete a researcher-created questionnaire on the frequency of parent–child engagement in 24 specific mathematics-related activities in the home (that fit into the categories of direct numeracy [10 items], indirect numeracy [seven items], and spatial activities [seven items]) by responding to the prompt “In the past month, how often did you and your child engage in the following activities?” with six options ranging from “never” (0), “one to three times per month” (1), “once a week” (2), “a few times per week” (3), “every day” (4), to “multiple times per day” (5; see Table 1 for descriptive statistics of all HME items). The questionnaire was based on previous research by LeFevre et al. (2009) and Hart et al. (2016). Specific items chosen from these prior scales were selected based on past performance and appropriateness for the preschool age level. Specifically, the research team did not include items (e.g., “wears a watch”) that were used in prior work, but had low engagement rates (i.e., mostly “never” was endorsed).

**TABLE 1:** Response rates, factor membership, and descriptive statistics for all home mathematics environment items.

Item number	Item description	% of parents responding “never”	*M*	*SD*
**Direct numeracy factor**				
1	Count objects	0.8	3.60	0.95
2	Print numbers	14.7	2.16	1.38
3	Read number storybooks	3.9	2.35	1.24
4	Use number activity books	14.7	1.86	1.28
5	Count down (10, 9, 8, 7…)	16.3	2.07	1.43
6	Learn simple sums (i.e., 2 + 2 = _)	32.6	1.40	1.29
7	Identify names of written numbers	23.3	2.05	1.46
8	Recite numbers in order	0.8	3.27	1.12
9	Use number flashcards	45.0	1.09	1.33
10	Note numbers on signs when driving or walking	20.9	1.95	1.50
**Indirect numeracy factor**				
11	Measure ingredients when cooking	20.9	1.78	1.30
12	Play board games with die or spinner (e.g., Chutes and Ladders, Trouble, etc.)	14.0	1.85	1.25
13	Talk about money when shopping (e.g., Which costs more?)	20.2	1.73	1.31
14	Play games that involve counting, adding or subtracting	17.1	1.81	1.24
15	Play card games that use numbers or counting (e.g., Go Fish, War)	29.5	1.47	1.35
**Spatial factor**				
16	Play computer/video games involving spatial tasks (e.g., Tetris)	27.9	1.67	1.43
17	Play with puzzles (such as picture puzzles, tangrams, slide puzzles, 3D puzzles)	8.5	2.40	1.30
18	Build with Legos, blocks, Lincoln Logs, or construction set (e.g., Duplo, Mega blocks, etc.)	5.4	2.74	1.38
19	Talk about location using terms such as in, on, under, around	3.1	3.01	1.20
20	Sort things by size, color or shape	6.2	2.56	1.27
21	Recognize shapes in the everyday world (signs, toys, blocks, games, etc.)	3.1	3.05	1.27
**Items not included in the model fitting analyses**				
22	Talk about math while watching sports (e.g., talk about the score, compare the scores, etc.)	55.6	0.78	1.08
23	Play with Dominoes	66.7	0.51	0.85
24	Draw maps/plans of buildings or locations	56.6	0.74	1.04

#### Numeracy Skills

The Preschool Early Numeracy Skills Screener – Brief Version (PENS-B; Purpura et al., 2015) was used to evaluate preschoolers’ numeracy skills. The PENS-B is a 24-item measure which assesses broad numeracy skills that children are exposed to in preschool and kindergarten. For all items, children are asked verbal questions. For some questions, children are shown a picture and asked about the picture (e.g., “Which box has the most dots?” while displaying a picture of four boxes of dots). Specific assessment areas include set comparison, numeral comparison, one-to-one correspondence, counting a subset, number order, numeral identification, ordinality, and number combinations. Children received one point for each correct answer. Although all 24 items were administered, a ceiling rule consistent with the measure development process (Purpura et al., 2015) was applied during analyses and children did not receive points for any correct responses after three consecutive incorrect responses. The PENS-B had high internal consistency (α = 0.88) for this sample.

#### Mathematical Language

The mathematical language assessment used was the Preschool Assessment of the Language of Mathematics (PALM; Purpura and Logan, 2015). The PALM is a 16-item measure of mathematics-specific language. Children were awarded one point for each correct response. In prior work (Purpura and Logan, 2015), these items were selected from a larger battery including a broader range of items using an item-response theory framework. The selected items had a range of difficulty parameters and strong discrimination parameters. The specific words included in this measure were intended to be broadly representative of the quantitative and spatial language associated with mathematics. Quantitative words included: *take away*, *a little bit*, *most*, *more*, *fewest*, and *less*. Spatial words included: *nearest*, *under*, *first*, *far*, *below*, *front*, *middle*, *end*, *last*, and *before*. All items were designed to be completed without exact quantitative skills and in a non-numeracy context. For example, the quantitative questions were asked in different ways: (a) comparing dots with such a gross difference that children would be able to respond correctly regardless of numeracy ability as long as they knew the meaning of the language terms (e.g., 10 vs. 2) and (b) using a picture of mostly full and mostly empty glasses when asking “Which glass has the most water?” or “Which glass has less water?” This mathematical language task had an internal consistency of 0.80 for this sample.

#### Spatial Skills

The spatial transformation task was from previous research by Levine et al. (1999). This task consisted of 32 problems, each involving a different target shape. On each problem, the child was shown two halves of a shape that had been divided along the vertical axis and was asked to “point to the picture the pieces make.” The child’s task was to select the whole shape from among four choices in a 2 × 2 array that could be formed from the halves. Four different forms of the task were used in this study. The forms varied in the positioning of the target pieces for a particular target shape. The 32 target shapes were randomly matched with one of the four different task forms. For example, target shape 1 used form (a) where the pieces were displayed in a horizontal translation configuration; target shape 2 used form (d) where the pieces were displayed in a diagonal rotation configuration; target shape 3 used form (b) where the pieces were displayed in a diagonal translation configuration; target shape 4 used form (c) where the pieces were displayed in a horizontal rotation configuration. This spatial task had an internal consistency of 0.76 for this sample.

#### Covariates

Child age, sex (male = 0, female = 1), and highest parent education (on a 9-point scale ranging from eighth grade or less to doctoral degree) were included as covariates.

### Procedure

#### Assessment Procedure

Assessments took place in the preschools in a room designated by the school directors or teachers. Assessments were administered in a counterbalanced order and were conducted across multiple sessions as needed. All assessments were conducted by graduate or undergraduate research assistants studying in social science fields. All assessors completed two 2- to 3-h training sessions and were required to demonstrate their competence and knowledge of assessments by “testing out” in order to participate in data collection. The testing out process involved administering each of the assessments to a lead project member who ensured that administration and scoring were done correctly.

#### Analytical Procedure

To identify the best-fitting factor structure of the HME, a series of seven confirmatory factor analyses (CFAs) were conducted in Mplus (Muthén and Muthén, 2012) largely in the same process as Hart et al. (2016). Before fitting the models, items with low usage were dropped from the data. These three items all had more than 50% of parents report they never engaged their children in these activities (see bottom of Table 1). Initially, a single factor CFA was fitted, encompassing all possible mathematics-related activities parents could engage in with their children in the home. Next, three 2-factor CFAs were fitted. The first 2-factor model had two factors representing direct numeracy (i.e., activities explicitly meant to teach children quantitative skills) and other activities (i.e., indirect numeracy and spatial items). The second 2-factor model had two factors representing spatial and other activities (direct numeracy and indirect numeracy items). The third 2-factor model had two factors representing indirect numeracy (i.e., activities associated with quantitative skills but not overt) and other activities (direct numeracy and spatial items). Then, a 3-factor CFA was fitted, with three factors representing direct numeracy, indirect numeracy, and spatial activities. Finally, following the process of Hart et al. (2016), we fit a bifactor model that included the specific factors of direct numeracy, indirect numeracy, and spatial environment, as well as a general HME factor that incorporated all items from the three specific factors. However, given model comparison results discussed later, we also fit an additional bifactor model similar to the 2-factor model that included a direct + indirect numeracy factor and a spatial factor, but that also included a general HME factor.

The bifactor model allows us to assess the overlapping variance among all the items (i.e., the general HME factor), as well as examine the remaining variance (i.e., residualized variance) that is specific to the types of home mathematics activities being conducted (e.g., direct numeracy, indirect numeracy, and spatial factors). This is done through regressing all items onto the general factor and the domain-specific items onto their specific domains and restricting the correlations between the factors to zero. Essentially, a bifactor model may provide a more precise measure of each specific factor by removing that which is common across the specific items. Critically, it allows us to better understand the domain-specific factors (i.e., direct numeracy, indirect numeracy, and spatial) after partialing out the general HME, as well as test whether the specific factors predict child outcomes over and above the general HME (Chen et al., 2006). For more detailed descriptions of bifactor models, see Reise (2012).

To compare model fit across the various models, the χ^2^ difference test was used to compare nested models where significant χ^2^ difference test indicates a worse fit for the more constrained model (i.e., in this instance, the model with fewer factors). Akaike Information Criterion (AIC) and sample-size adjusted Bayesian Information Criterion (BIC) were used to evaluated relative model fit of all models. Lower AIC and BIC values—typically differences of 10 or more—indicated a better fitting model (Kass and Raftery, 1995; Hu and Bentler, 1999; Burnham et al., 2011). Once the best-fitting factor model was determined, an item dropping process was conducted to remove poor fitting items (specifically, items that either did not load or loaded negatively on one of the sub-factors). Finally, a structural equation model was conducted to investigate the relations of the individual factors with measures of numeracy, mathematical language, and spatial skills, controlling for children’s age, sex, and parent education. As there was some missing data on some of the direct assessments (4.7 to 7.0%), full information maximum likelihood was used in the analyses.

## Results

Descriptive statistics for key study variables can be found in Table 2. Correlations between the final latent factors and all outcome variables (i.e., numeracy, mathematical language, and spatial skills) are presented in Table 3.

**TABLE 2:** Descriptive statistics for key demographic variables and direct assessments.

	*N*	*M*	*SD*	Observed range	Skewness	Kurtosis
Age (years)	129	4.71	0.55	3.07–6.03	−0.29	0.12
Parent education	129	6.07	1.93	2–9	−0.07	−1.01
Numeracy skills	121	12.57	5.93	0–24	−0.27	−0.82
Mathematical language	120	12.34	3.08	1–16	−1.21	1.06
Spatial skills	123	13.08	5.12	4–27	0.41	−0.53

**TABLE 3:** Correlations between home mathematics environment factors and direct assessments.

	1	2	3	4	5	6
1. Direct + indirect numeracy	–					
2. Spatial	0.00	–				
3. HME	0.00	0.00	–			
4. Numeracy skills	0.36***	0.00	−0.20*	–		
5. Mathematical language	0.36***	0.04	−0.21*	0.61***	–	
6. Spatial skills	0.25**	0.13	0.01	0.45***	0.27***	–

HME, home mathematics environment. * p < 0.05, ** p < 0.01, *** p < 0.001.

### Evaluating the Factor Structure of the Home Mathematics Environment Items

The first goal of this study was to examine the factor structure of the HME. The fit statistics for the five initial models (1-factor, three 2-factor, 3-factor) are displayed in Table 4. Overall, none of the models tested provided an excellent fit to the data according to fit indices (SRMR ≤ 0.10, CFI and TLI ≥ 0.90, RMSEA ≤ 0.08; Hu and Bentler, 1999). Among these models, the 2-factor model that combined direct numeracy and indirect numeracy (Model 3) was a better fit to the data than either of the other 2-factor models (compared to Model 2, ΔAIC > 10, ΔBIC > 10; compared to Model 4, ΔAIC > 10, ΔBIC > 10) and the 1-factor model (Δχ^2^ = 37.48, *df* = 1, *p* < 0.001; ΔAIC > 10, ΔBIC > 10). Moreover, it did not significantly differ in fit from the 3-factor model (Δχ^2^ = 3.60, *df* = 2, *p* = 0.165; ΔAIC < 10, ΔBIC < 10). This is likely because of the very high correlation between the direct numeracy and indirect numeracy factors (*r* = 0.93), whereas the correlations between the direct numeracy factor and the spatial factor (*r* = 0.72) and the indirect numeracy factor and spatial factor (*r* = 0.77) were, though still high, more differentiable. Given these findings, two bifactor models were analyzed—one 3-factor bifactor model and a 2-factor bifactor model. The 2-factor model included a factor that combined direct and indirect numeracy items, a spatial factor, and a general HME factor (this model was aligned with Model 3, but also included the general HME factor). As can be seen in Table 4, the two bifactor models did not fit significantly differently (ΔAIC < 10, ΔBIC < 10), but both demonstrated better fit indices than the non-bifactor models (ΔAIC and ΔBIC > 10 for all model comparisons). Thus, for parsimony, the 2-factor bifactor model was selected as the preferred model. After selection of the 2-factor bifactor model, and because the model fit indices did not consistently indicate an excellent fit to the data, an exploratory model fitting approach was conducted to improve overall model fit that aligned with the process in Hart et al. (2016). This was done because we intended to use this model in further structural equation modeling in the second research goal. Items that either did not significantly load onto one of the specific factors in the bifactor model, or that negatively loaded onto a factor were removed from the final model because this would indicate that the item does not provide any information for the specific factor on which it was theoretically placed.

**TABLE 4:** Model fit indices for each tested model representing the home mathematics environment.

#		*X*^2^	*df*	*p*	AIC	Adj. BIC	RMSEA	RMSEA lower bound	RMSEA upper bound	CFI	TLI	SRMR
**Initial models**												
1	1-Factor home mathematics environment	479.31	189	<0.001	8544.72	8724.89	0.11	0.10	0.12	0.68	0.64	0.09
2	2-Factor IHNE + spatial vs. DHNE	466.83	188	<0.001	8534.24	8514.86	0.11	0.10	0.12	0.69	0.66	0.09
3	2-Factor DHNE + IHNE vs. spatial	441.83	188	<0.001	8509.24	8489.86	0.10	0.09	0.12	0.72	0.66	0.09
4	2-Factor DHNE + spatial vs. IHNE	476.19	188	<0.001	8543.60	8726.63	0.11	0.10	0.12	0.68	0.65	0.09
5	3-Factor DHNE, IHNE, and spatial	438.23	186	<0.001	8509.64	8489.65	0.10	0.09	0.12	0.72	0.69	0.09
**Bifactor models**												
6	3-Factor bifactor solution	337.95	168	<0.001	8445.35	8419.91	0.09	0.08	0.10	0.81	0.77	0.08
7	2-Factor bifactor DHNE + IHNE vs. spatial	334.50	168	<0.001	8441.90	8416.46	0.09	0.07	0.10	0.82	0.77	0.07
**Final model**												
**7a**	**2-Factor bifactor DHNE + IHNE vs. spatial with 10 items removed**	**51.53**	**33**	**0.021**	**4465.49**	**4452.17**	**0.07**	**0.03**	**0.10**	**0.95**	**0.92**	**0.05**
**Model checks**												
8	2-Factor DHNE + IHNE vs. spatial with 10 items removed	87.92	43	<0.001	4481.88	4471.59	0.09	0.06	0.12	0.87	0.85	0.08
9	1-Factor with 10 items removed	176.25	44	<0.001	4568.21	4558.22	0.15	0.13	0.18	0.66	0.58	0.11

*DHNE, direct home numeracy environment; IHNE, indirect home numeracy environment; spatial, spatial environment. Bolded model indicates the final, best-fitting model, a 2-factor bifactor model that consists of direct + indirect numeracy, spatial, and general home mathematics environment factors, after adjusting item selection.*

**FIGURE 1:**
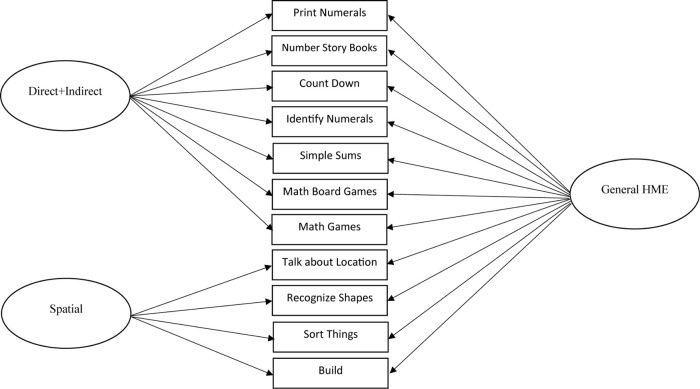
Best-fitting model, the final 2-factor bifactor model (direct + indirect numeracy, spatial, and a general home mathematics environment [HME] factor).

The initial step of item dropping resulted in 10 items being dropped, eight for non-significant loadings onto the direct + indirect numeracy factor (Items 1, 4, 8, 9, 10, 11, 13, and 15), and two for non-significant loadings onto the spatial factor (Items 16 and 17). Although modification indices suggested that the model could be improved by loading Item 8 (recite numbers in order) onto the spatial factor, the item was removed as it did not logically fit on the spatial factor and did not significantly load onto the direct + indirect numeracy factor. Although the removal of these 10 items resulted in two more items being non-significant on their factors (Item 6 on the general HME factor and Item 18 on the spatial factor) when the model was rerun, additional reduction of items resulted in models where the residual covariance matrix was not positive definite. Thus, the model that included Items 6 and 18 was determined to be the final model. This resulted in an excellent fitting model (7a) as can be seen in Table 4 in bold font. The final model included 11 items (seven on the direct + indirect numeracy factor and four on the spatial factor). All factor loadings for the direct + indirect numeracy, spatial, and general HME factors can be seen in Table 5, with the model displayed in Figure 1. To ensure that the item dropping process did not alter the model structure, we conducted two subsequent model checks. Using the final selected items, we reran Models 1 and 3 (1-factor and 2-factor direct + indirect numeracy models) without the 10 items that were dropped. These resulted in Models 8 and 9 (see Table 4). Neither of these models fit better than the 2-factor bifactor model with the 10 items dropped (i.e., Model 7a; ΔAICs > 10, ΔBICs > 10). The 3-factor model (Model 5) was not rerun because only two indirect numeracy items were retained which would have been insufficient to run the model.

**TABLE 5:** Standardized factor loadings from the final, best-fitting model, a 2-factor bifactor model.

	Direct + indirect numeracy	Spatial	General home mathematics environment
Print numerals	0.44	—	0.34
Number story books	0.29	—	0.52
Count down	0.55	—	0.34
Identify numerals	0.64	—	0.23
Simple sums	0.70	—	0.18
*Math board games*	0.39	—	0.37
*Math games*	0.34	—	0.67
Talk about location	—	0.72	0.27
Recognize shapes	—	0.45	0.65
Sort things	—	0.42	0.69
Build	—	0.23	0.44

*All items significantly loaded onto each factor with the exceptions of the “simple sums” item which did not significantly load on the general home mathematics environment factor and the “build” item which did not significantly load on the spatial factor. However, both were retained in the model as removing them resulted in models in which the residual covariance matrices were not positive definite. Italicized items were originally on the indirect factor.*

### Do the Home Mathematics Environment Factors Predict Preschoolers’ Numeracy, Mathematical Language, and Spatial Skills?

**FIGURE 2:**
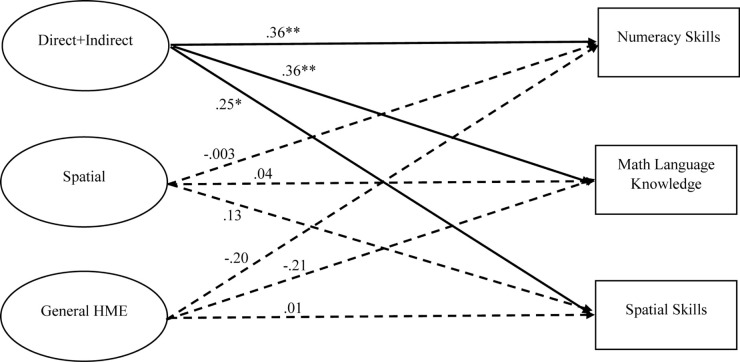
Relations between home numeracy environment factors and direct assessments of children’s numeracy skills, mathematical language knowledge, and spatial skills. Standardized coefficients are presented in the figure. *p < 0.05, **p < 0.01.

Correlations for key HME factors and direct assessments can be found in Table 3. A structural equation model was used to examine how parent–child home mathematics activities were associated with children’s mathematics skills (see Figure 2). The direct assessments of children’s numeracy, mathematical language, and spatial skills were regressed on the three HME factors (general HME, direct + indirect numeracy, and spatial) as well as covariates (age, sex, parent education). The model fit statistics were good (CFI = 0.91, TLI = 0.87, RMSEA = 0.07, SRMR = 0.07). Among the covariates, both age and parent education significantly predicted all three direct assessments; sex was not a significant predictor of any of the three direct assessments. Overall findings suggest that only the direct + indirect numeracy factor significantly predicted child performance on numeracy (β = 0.36, *p* = 0.004), mathematical language (β = 0.36, *p* = 0.001), and spatial skills (β = 0.25, *p* = 0.022). Neither the general HME factor nor the spatial factor were significant predictors of any of the three direct assessments.

### *Post hoc* Analyses

These findings may suggest that, even though the bifactor model is the one that best represents the structure of the HME, only the direct + indirect numeracy factor is important in uniquely predicting child outcomes, which raises the issue of whether the bifactor structure is necessary. To address this issue, we conducted *post hoc* analyses using just the direct + indirect numeracy factor (and covariates) in predicting the three direct assessments. These results indicated that, without the bifactor model, the direct + indirect numeracy factor was only significantly related to children’s spatial skills (β = 0.22, *p* = 0.015) and not numeracy (β = 0.15, *p* = 0.088) or mathematical language (β = 0.16, *p* = 0.084). Similar results are found when just using the general HME factor as a predictor (with covariates in the model), in which the general HME factor was only significantly related to children’s spatial skills (β = 0.19, *p* = 0.028) and not numeracy (β = 0.06, *p* = 0.522) or mathematical language (β = 0.07, *p* = 0.438). These supplemental analyses suggest that the bifactor structure may be necessary in understanding how the HME is related to children’s skills because it provides a “more pure” measure of the specific factors (i.e., with the bifactor model it is measuring what is unique to the specific direct + indirect numeracy factor after removing what is more general to the HME).

## Discussion

A growing body of research has begun to examine the relation between the HME and children’s mathematics performance. However, much of this literature utilizes models of the HME that are based on prior work, but does not explicitly test the measurement models within their specific study. Moreover, few studies empirically contrast multiple models found in prior literature. The first objective of the present study was to address this limitation by attempting to replicate the factor structure of the HME (Hart et al., 2016) in a different sample by comparing it to several alternative, but plausible models. The second objective in this study was to extend the findings of Hart et al. (2016) to examine the relations between the identified HME factors and direct assessments of children’s numeracy, mathematical language, and spatial skills (as opposed to only using parent reports of children’s mathematics and spatial skills as was done in Hart et al., 2016). In contrast to Hart et al. (2016), who found a 3-factor bifactor model (general HME, direct numeracy, indirect numeracy, and spatial), we found that a 2-factor bifactor model (general HME, direct + indirect numeracy, and spatial) was the more parsimonious model. Moreover, whereas Hart et al. (2016) found that the general HME factor was the aspect of the HME that was related to parent reports of children’s mathematics performance, we found that the direct + indirect numeracy factor was the only aspect of the HME that was related to direct assessments of children’s numeracy, mathematical language, and spatial skills. Although the current findings diverge from the findings of Hart et al. (2016), they both suggest the importance of including the bifactor structure. The differences between specific predictors in the current study and the Hart et al. (2016) study may be a result of a number of reasons discussed below. Importantly, the *post hoc* analyses indicated the bifactor structure was necessary to understand the link between the HME and children’s mathematics skills because it allows for a more precise estimate of the specific factors than models that do not include the bifactor structure.

### The Home Mathematics Environment Factor Structure

The bifactor model with an overarching HME factor and specific factors of direct + indirect numeracy and spatial skills is largely similar to the model proposed by Dearing et al. (2012) suggesting that numeracy and spatial factors separate into distinct categories rather than more refined categories within those areas (e.g., direct vs. indirect). This may be due to the nature of direct versus indirect items included in these models. For example, the types of indirect activities that loaded on the direct + indirect numeracy factor (those items italicized in Table 5) were primarily game-based mathematics activities where there is likely an intentionality of focusing on mathematics during the games (e.g., mathematics games) or even if there is no intentionality in explicitly teaching mathematics during the games, there are ample opportunities for mathematics-related discussions to arise (e.g., mathematics-related board games). As these were the types of indirect items retained in the final models, it may indicate that measurement of the HME, when specifically referring to the numerical component, must center on activities where there is direct intentionality of teaching mathematics, or where the opportunities of engaging with mathematics content are explicit. Notably, other indirect numeracy items where the numerical content was not as explicit (e.g., measuring ingredients) were dropped from the model as they did not contribute to the direct + indirect numeracy factor. An additional important result of the item reduction process was that the HME was effectively measured through only a relatively small number of items (seven total items for the direct + indirect factor and four spatial items). The small number of items may support enhanced feasibility of collecting similar data in future studies as parents would not have to complete extensive surveys.

Building upon the work of both Dearing et al. (2012) who suggested a numeracy versus spatial activities factor structure and Hart et al. (2016) who incorporated a bifactor structure, the bifactor framework in the current study was also found to be the best fit for the data. The bifactor model allows for the items from all the specific factors to load onto a more general factor that captures the general variance in the HME. Importantly, the specific factors (direct + indirect numeracy, spatial) represent the unique variance from the factor-specific items that were not accounted for (i.e., the residualized variance) on the general HME factor. The specific factors from the current bifactor model differ slightly from the specific factors in the bifactor model from Hart et al. (2016) as they found the direct and indirect numeracy factors to be separable. The consolidation of the direct and indirect factors in the current study was not surprising given the high correlation between these two factors in the original three factor model (Model 5). This relation could be due to the specific types of indirect activities that were included (e.g., talk about money when shopping, play games that involve counting) because, even though they are indirect numeracy activities, parents may engage in them with their child intentionally to support mathematics skills which would effectively make them a more direct activity. Future work should examine parent intentionality in engaging in indirect activities and how that may affect the association with direct activities.

The inclusion of the bifactor structure in the current model enables us to parse out the aspects of the HME that are more general to parent–child mathematics interactions and those that are construct specific. This may reflect an intentionality of focus (engaging in explicit and directed activities focused on mathematics) for the specific factors versus broad engagement for the general HME factor. Alternatively, the general HME factor could capture the variance that is more general to the overall home learning environment (even beyond mathematics) and the specific factors may account for the mathematics-specific variance. However, more work is needed to explicitly test these assumptions. In particular, work that extends domain-specific home environment evaluations to examine the structure of multiple facets of the home environment—including mathematics, literacy, and self-regulation—will enable researchers to better understand the domain-general and domain-specific aspects of the home environment that support children’s learning.

### Relations Between the Home Mathematics Environment and Preschool Numeracy, Mathematical Language, and Spatial Skills

After identifying the HME factor structure, we examined the extent to which the HME predicted direct assessments of preschoolers’ numeracy, mathematical language, and spatial skills. The findings suggest that the direct + indirect numeracy aspect of the HME is an important predictor of children’s performance, in line with previous research (Blevins-Knabe and Musun-Miller, 1996; Kleemans et al., 2012). Specifically, results from the structural equation model indicated that the direct + indirect numeracy factor positively predicted direct observations of preschoolers’ numeracy, mathematical language, and spatial skills, but the spatial environment factor did not predict any of the outcomes. This may be because of the type and frequency with which activities occur in the spatial environment. Notably, most of the spatial items in the final spatial factor are ones that occur with high frequency (means of around “a few times per week” which was relatively high compared to other types of items), but also are activities in which children may do more on their own than in an interactive setting with adults (e.g., sorting, building). Thus, even though children may engage in these activities, they may not be receiving the feedback and scaffolding necessary to develop these targeted skills as would be found with more guided or interactive play opportunities (Toub et al., 2018). Thus, these findings do not suggest that the home spatial environment activities are not a valid target for future assessment or intervention, but rather that simply measuring the quantity of this type of play may not be sufficient for linking it to children’s mathematics or spatial performance. Given that other studies (Dearing et al., 2012; Zippert and Rittle-Johnson, 2018) also did not find specific relations between the home spatial environment and children’s spatial skills, but studies that measure the direct engagement of parent–child spatial language do demonstrate relations (Ferrara et al., 2011), future research should extend this work to examine the quality of these activities and parents’ explicit focus on spatial properties during such activities, as well as additional factors such as parent spatial skills that has previously been found to be related to children’s performance (Zippert and Rittle-Johnson, 2018).

Importantly, though previous work (Napoli and Purpura, 2018) found a link between the HME and children’s general vocabulary, this is the first study to establish a link between the HME and children’s mathematical language skills. The parent–child interactions that occur during HME activities may involve specific uses of mathematical language terms. For example, if parents and children are counting, a parent may ask “What number comes *next*?” or “What is the number *after* four?” When playing mathematics games with their children, parents may ask, “Who has *more* [or *the most*] points?” Knowledge of these terms and concepts may be supported through HME interactions. Conversely, as these data are concurrent, it is also possible that the directionality of the relation is such that children’s knowledge of mathematical language supports engagement in HME activities. Specifically, knowledge of mathematical language terms and concepts may provide children with access to understanding the concepts presented through the HME which, then, may enable the HME to support children’s numeracy development. Moreover, it may be that children who know mathematical language terms and concepts may prompt more parent initiations of mathematics activities. Future longitudinal research that addresses the potential mediational role of mathematical language should be conducted to better examine these mechanisms. Moreover, it is important to highlight that all the direct assessments, but particularly mathematical language and numeracy, were significantly related. These strong relations may potentially explain why the direct + indirect factor was related to all direct assessments. It may be that when parents engage their children in mathematics-focused activities, they may go beyond just explicit teaching and also use significant amounts of math talk (Eason and Ramani, 2020). It is possible that this math talk may expand beyond simple numeracy-related talk to include mathematical language and spatial skills; however, the specific types of talk parents use during these types of activities at home needs to be further investigated.

The current findings contrast with Hart et al. (2016) in that the general HME factor was not the factor that was related to children’s performance. This may be because the age range measured in Hart et al. (2016) was twice as large as the age range in the current study (3 to 8 versus 3 to 5 years old). With the broader age range, the specific skills associated with the direct + indirect numeracy factor may not have been as indicative of performance because different HME indicators have been found to be differentially related to performance at different ages (Thompson et al., 2017). Thus, the general HME factor may have been measuring more of the overarching mathematics-related practices. The relation between the general HME factor and mathematics performance in Hart et al. (2016) may be because the general factor was accounting for important variance across the items that were more general across ages. In contrast, with the narrower age range in the current study, the direct + indirect numeracy factor may have been capturing more of the specific skills associated with children’s mathematics development at this age while the general HME factor may have simply been capturing the broader environment that may not necessarily be specific to mathematics performance. Alternatively, this could be indicative of developmental change in the functioning of the HME in that a more explicit and intentional focus may be necessary during the younger years, whereas a broader supportive environment may be important as children are in early elementary school. However, it should be cautioned that these age-related hypotheses cannot be evaluated through the current study as the age range is more narrow than the Hart et al. (2016) study and that further work explicitly testing these hypotheses and disentangling potential age-related differences in these models is needed.

### Limitations and Future Directions

The present study should be considered within the context of specific limitations. Similar to Hart et al. (2016) the reliance on parent-reported HME may have biased the results if parents indicated higher frequencies for HME activities due to social desirability. However, there were a few activities that were rated by many parents as infrequent, suggesting that response bias may not be a large concern. Our view of the HME was also limited by having one parent reporting on the home environment (mostly mothers). Parents may individually and uniquely contribute to children’s home environments, which we could not capture with a single parent reporting in instances where there are two parents or caregivers in the home. Additionally, it may be plausible that the HME may act as a proxy for genetics and parent mathematics skills, given that children’s genes for mathematics skills are correlated with their home environment and there is evidence of some genetic influence on different aspects of mathematics performance (Plomin et al., 1977; Scarr and McCartney, 1983; Hart et al., 2016). However, the data needed to test for a gene–environment correlation or account for parent mathematics skills were not available in the current study. Future research on the HME should account for gene–environment correlations. Similarly, children with greater mathematics skills (i.e., numeracy, mathematical language, or spatial skills) may elicit or initiate a greater number of mathematics-related interactions in the home; however, given the cross-sectional nature of this data we cannot test the directionality of the association (Hart et al., 2019). Thus, future research should consider using longitudinal data to test the directionality of the association between the HME and children’s mathematics skills. Furthermore, the CFA was limited by a small sample size. Specifically, a weakness of utilizing a bifactor model is the prevalence of over-extraction which is compounded by small sample sizes (Rindskopf, 1984). Future research should replicate the current study’s CFA with a larger sample size. Furthermore, inclusion of only a core set of mathematics-related skills (numeracy, mathematical language, and spatial skills) that have most strongly been linked with mathematics development more broadly were included in the study. Future work should consider a broader range of HME facets (e.g., patterning, geometry) such as was done by Zippert and Rittle-Johnson (2018) and their connected skills. Subsequent work should also use multiple measures of each of the child assessments to reduce measurement bias. Finally, both quantitative and spatial language were included in the mathematical language measure, and it is unclear if the home spatial factor would have been related to a measure of spatial language that was independent of quantitative language. As it is not possible to disentangle these types of mathematical language in the current study, future work should examine the factor structure of mathematical language and if distinct aspects of the HME are uniquely related to the various aspects of mathematical language.

## Conclusion

In conclusion, we worked to replicate and extend previous work by separating the HME into direct + indirect numeracy and spatial components with an overarching general HME factor, and testing these factors’ associations with preschoolers’ numeracy, mathematical language, and spatial skills. The results indicate that only the direct + indirect numeracy factor predicted preschoolers’ specific mathematics skills, highlighting the importance of parent–child engagement in specific aspects of mathematics-related activities.

## Data Availability Statement

The datasets for this study will not be made publicly available because we are not allowed to share data outside the key personnel for the project by our IRB. Requests to access the datasets should be directed to the corresponding author.

## Ethics Statement

The studies involving human participants were reviewed and approved by Purdue University Institutional Review Board. Written informed consent to participate in this study was provided by the participants’ legal guardian/next of kin.

## Author Contributions

DP, YK, ER, SS, SH, and CG contributed to the conceptualization and design of the study. DP, ER, and SH contributed to data analysis and results. All authors contributed to the writing of the manuscript.

## Conflict of Interest

The authors declare that the research was conducted in the absence of any commercial or financial relationships that could be construed as a potential conflict of interest.
